# Ethnic markers and the emergence of group-specific norms: an experiment

**DOI:** 10.1038/s41598-022-07981-z

**Published:** 2022-03-24

**Authors:** Juan Ozaita, Andrea Baronchelli, Angel Sánchez

**Affiliations:** 1 Grupo Interdisciplinar de Sistemas Complejos (GISC), Departamento de Matemáticas, Universidad Carlos III de Madrid, 28911 Leganés, Madrid Spain; 2 Department of Mathematics, City, University of London, London, EC1V 0HB UK; 3 The Alan Turing Institute, British Library, 96 Euston Road, London, NW12DB UK; 4 Instituto de Biocomputación y Física de Sistemas Complejos (BIFI), Universidad de Zaragoza, 50018 Zaragoza, Spain

**Keywords:** Scientific data, Human behaviour

## Abstract

Visible markers are an important factor in social interactions. Some researchers have argued that one of their roles is to promote cooperation, but models designed to address this issue have yielded contradictory results. Here we present an experimental study of the effect of visible markers on the emergence of social norms where human subjects play a binary coordination game. Our results do not show different, marker-dependent behaviors. Instead, in practically all sessions participants achieved a global equilibrium disregarding the markers. Our findings suggest that simple markers may have a limited role in promoting the emergence of group-dependent social norms and call for further research investigating the role of markers in more sophisticated social settings.

## Introduction

A factor to understand cooperation in large societies is the analysis of the social norms regulating interactions between individuals^1,2^. Among different cultures, collective commitments allow to begin and sustain cooperation between groups of individuals. In large societies, these agreements are specific and more complex. Culture coordinates the values and behaviors that are transmitted within a society, manifesting itself in features like dress, cuisine, language, wealth and status symbols, etc.^3^ In order to be self-sustained, these cultural features need to be visible^4–6,8^, so every individual can identify themselves and others into some collective category.

In this context, several researchers have argued that external visibility, in particular, arising from ethnic markers that signal ethnic group membership, provides a loci for cooperation^10–12^, enabling individuals to create judgements and make decisions based on the information of those external features. In order to model this behavior, different dynamics have been used, especially from the perspective of game theory. A key work along this line was carried out by McElreath et al.^11^, who introduced a mathematical model, based on an evolutionary coordination game, showing that groups exhibiting differences in both social norms and ethnic markers can emerge and remain stable under rather general conditions. This kind of correlations would represent what we may call collective agreements. One of the conditions for their emergence was that individuals acquire markers and behaviors by imitating successful ones. Importantly, in the model proposed in Ref.^11^, there is no initial correlation between markers and behavior, i.e., the correlation between marker and behavior resulting from the evolution is a truly emergent phenomenon.

In reality, agents may not be able to gather information from others’ payoffs and, therefore, they may not identify the individual and behavior to imitate. This possibility led us to study an alternative dynamics based on reinforcement learning dynamic for every agent^13–17^. This type of evolutionary dynamics makes agents choose their behavior based only on the reinforcement or avoidance of actions leading to improving their payoff, without making reference to any external agent. Our results, also based on binary coordination games and binary markers^7^ confirmed that correlations between external visible features (called markers in our model) and behavior can arise for all the population, similarly to the collective agreements mentioned above. However, we observed this alignment between behavior and markers for a somewhat restricted set of parameters. If there is an important gap between coordinating and non-coordinating in terms of payoff, it could be more efficient to stick to one option always, independently of the external visible marker. The energy that costs producing different strategies for every different situation (depending on the visible marker), can be balanced with the accuracy that can be obtained from a single strategy.

The fact that the evidence for correlation between behaviors and markers is not really conclusive, along with the contradictory results of former experimental tests^18–22^, calls for further experiments specifically designed to address this issue. In this paper, we undertake this task by carrying out experiments closely mimicking the models in Refs.^7,11^. The idea is to analyze the data obtained from these experimental sessions and the type of equilibria that arise, comparing it to the theoretical models to assess whether or not marker-dependent behavior arises. This translates into a set of specific, preregistered^23^ hypotheses which we will introduce below when discussing our results. In the final part of the paper we will summarize the main results and compare them to these hypothesis. Details about the experiment can be found in the Methods section, including a translation of the experimental instructions.

## Results

### Experimental setup

The core of the experiment is organized around a coordination game, which participants played against a randomly chosen opponent that changed every round. Every participant is assigned a virtual “hat”, blue or yellow, at the beginning of the experiment, and the opponent’s hat is made known to her prior to making a new decision in the coordination game. In accordance with both McElreath et al.’s model^11^ and our model^7^, we did not give any direction to individuals to use one specific behavior or another depending on the marker, as we wanted to study whether even in this scenario behavior-marker correlation can arise as theoretically predicted. In other words, we are interested on the possibility of marker-dependent behavior arising spontaneously and not on its stability once it exists. We designed a 2 $\times 2$ experimental setup involving two separate treatments:

- Homophily, or tendency towards individuals with the same external visible features as the subject. We chose to study two different configurations: populations without homophily (agents interact with all agents with the same frequency) and populations with homophily (agents interact with their marker partners three times more than agents with the other marker). This choice was inspired by the original results of McElreath et al.^11^, who concluded that correlations between marker and behavior were favored if people preferentially interact with others with their same marker. In contrast, in^7^ we did not observe any special effect from this factor. We considered two scenarios, interactions occurring with equal probability irrespective of the opponent’s hat, and a biased scenario in which the opponent had the same hat as the focus individual with probability $75\%$.
- Group size. In our model based on reinforcement learning, we found that the size of the group does not change the qualitative behavior of the population. However, we also found the dynamics of the process may depend on the group size, the emergence of behavior correlated with the markers being more or less abrupt. Learning more about this motivates this choice for the second treatment.The original model studied several groups among which individuals can move (migration), but here we will focus on a single group, thus ignoring migration effects. We considered populations of *N* = 10 and *N* = 20 individuals.

The coordination game is defined through a 2 $\times$ 2 symmetric payoff matrix, where two identical actions are rewarded, and two different actions are punished. Thus, we can define it as :

1 $$\begin{aligned} \pi = \begin{pmatrix} 1 &{} -0.5\\ -0.5 &{} 1 \end{pmatrix}. \end{aligned}$$

In order to check the specific setup of the different experimental sessions, please check the methods section.

### Theoretical model on ethnic markers

We recall here the theoretical model of reinforcement learning with ethnic markers. The population plays a binary coordination game with two actions and one external visible marker with two different possibilities. Agents interact in a coordination game, reinforcing every action that succeeds and making less likely the ones that fail. Success or failure refers to the payoff being, respectively, larger or smaller than the agent’s aspiration threshold, which is an idiosyncratic quantity, an individual threshold that separates a positive from a negative outcome given a certain payoff. The stimulus is then defined as a bounded quantity, between $-1$ (maximum rejection) and 1 (maximum reinforce). The probability of choosing an action ($p_{a,t}$) at a time $t+1$ is related with a stimulus ($s_{a,t}$) computed from the difference between the aspiration level and the payoff at time *t*:

2 $$\begin{aligned} p_{a,t+1}= \left\{ \begin{array}{lcc} p_{a,t}+(1-p_{a,t})ls_{a,t} &{} \mathrm{if} &{} s_{a,t}\ge 0, \\ \\ p_{a,t}+p_{a,t}ls_{a,t} &{} \mathrm{if} &{} s_{a,t}<0. \\ \end{array} \right. \end{aligned}$$

The stimulus is calculated as the difference between the payoff ($\pi _{a}$) and the aspiration level (A), normalized by the maximum difference between the aspiration level and the payoff matrix^14^. This is:

3 $$\begin{aligned} s_{a} =\dfrac{\pi _{a}-A}{sup[|T-A|,|R-A|,|P-A|,|S-A|]}, a \in C,D. \end{aligned}$$

Besides this individual evolution of the dynamics, it is interesting to study populations of these agents. When the aspiration values are in a certain range of values, global strategies appear that may be different depending on the markers of the players. In the model, there are also external parameters that influence the distribution of interactions for each agent. These are the group size (*N*), which defines the size of the subpopulations that can interact, and the homophily (*e*), that defines, for each individual, which proportion of their interactions will be with agents that do not share a certain feature (the marker) with them. Therefore, a value of $e=1$ means total independence to be paired with someone with any marker, and $e=0.5$, means that a $50\%$ of the interactions will be with agents that share the same marker as the subject.

### Experimental results

As shown in Ref.^7^, the main conclusion from the above model is that global agreements which may be marked arise in populations of reinforcement learning agents under certain conditions on the parameter values. Those parameters, such as the aspiration level or the learning rate [*l* in Eq. (2)] are properties of the agents, and it is not possible to know their values in a population. Therefore, we have undertaken these experiments in order to assess whether, in the absence of such knowledge, the behavior predicted by the model can still be observed among real human subjects.

We analyzed the experimental results following our pre-registered set of hypotheses. Starting from a general prediction about the behavior of players of the coordination game, namely that coordination would be mediated by markers, we formulated the following set of specific hypotheses:^23^

**H1**: A correlation is established between the marker of the opponent and the action the subject chooses, meaning that the final equilibrium strategy for an agent may include different actions depending on the marker of the opponent.
**H2**: Homophily, understood as a selection bias towards agents with the same marker, increases the creation velocity of the intra-marker correlation, this is, the strategy for individuals that share marker.
**H3**: The correlation can only exist if a majority of the group share a preference order such that a coordination outcome is more desirable than an uncoordination one. We say that an outcome is more desirable when it has a higher value in a 1 (very pessimistic) to 7 (very optimistic) scale.
**H4**: Correlation can only be established if the former preference order can be seen as stationary, this is, it does not evolve in time or evolves in a slow time scale.
**H5**: A correlation is established regardless the size of the group, but its creation velocity is directly proportional to it.
**H6**: Individuals with a higher degree of norm compliance will arrive at a correlation between opponent’s marker and action chosen faster.
**H7**: Groups with lower overall degree of norm compliance will take longer to evolve correlations between markers and actions.
**H8**: Individuals will be more influenced by others who share their marker when making decisions.

**Figure 1:**
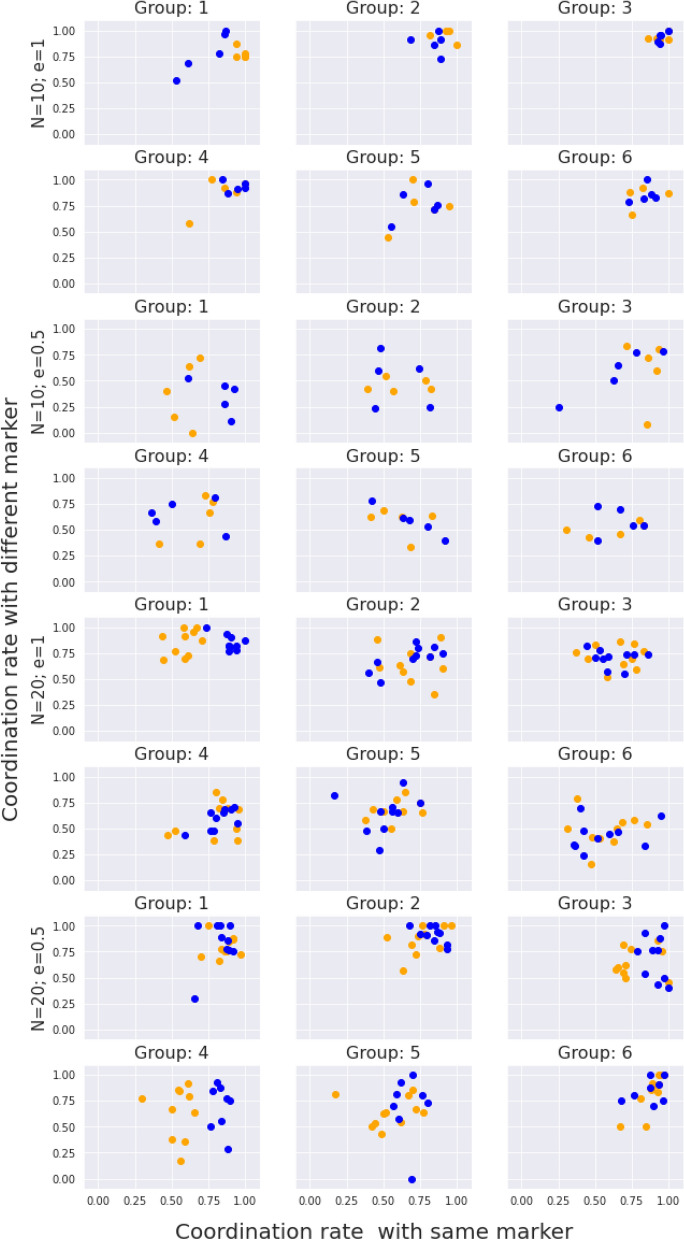
Experimental profiles of coordination rates for the transient(rounds 0 to 40); X-axis: coordination rate within the subpopulation that shared the same marker as the individual; Y-axis: coordination rate within the subpopulation that shares the complementary marker. Coloured, the marker of the participants.

Coming from the coordination game described in the methods section, we proceed to analyze its dynamics, considering the existence of a possible difference between the interactions with someone that shares the marker with the subject. In order to understand this coordination dynamics in the different experimental sessions, we have resorted to a 2D scatterplot (Fig. 1), where the horizontal axis represents the probability of coordinating with someone with the same marker and in the vertical axis we show the same quantity for the other marker. We have calculated this probability in a frequentist manner, defining it as the number of successful coordinations divided by the number of interactions for each of the cases. We have restricted the computation to the first forty rounds in order to allow for a more clear visualization. The different colors represent the two different markers. Figure 1 gives us information about hypotheses **H1**, **H2** and **H5**, i.e., those related with the structure of the collective equilibrium. Of course, the key hypothesis is **H1**, so we begin the discussion with it. As can be seen from the plot, although there are a couple of cases with marked collective strategies (cf. group 1 for $N=20$, $e=1$ and group 4 for $N=20$ and $e=0.5$), the general result is that players in a population tend to gather in a unique cluster, exhibiting behavior that ignores the markers. Otherwise, no clear pattern appears, which leads us to conclude that hypothesis **H1** is not correct.

As a global unique consensus is reached in the majority of the groups in our experimental sessions, the question arises of the role of herding behavior arises. In order to check the relevance of this factor, we made a similar plot to Fig. 1, but instead of using the coordination with the same and different marker as horizontal/vertical axis, we used the coordination with the most popular option/least popular option in a group. This most popular option is defined through the mode of the actions in a group for all the dynamics of the game. We can then track whether the coordination towards a single equilibrium is related to a single option chosen from the beginning, or it is the result of the evolution of two different marked consensus converging into a single one.

**Figure 2:**
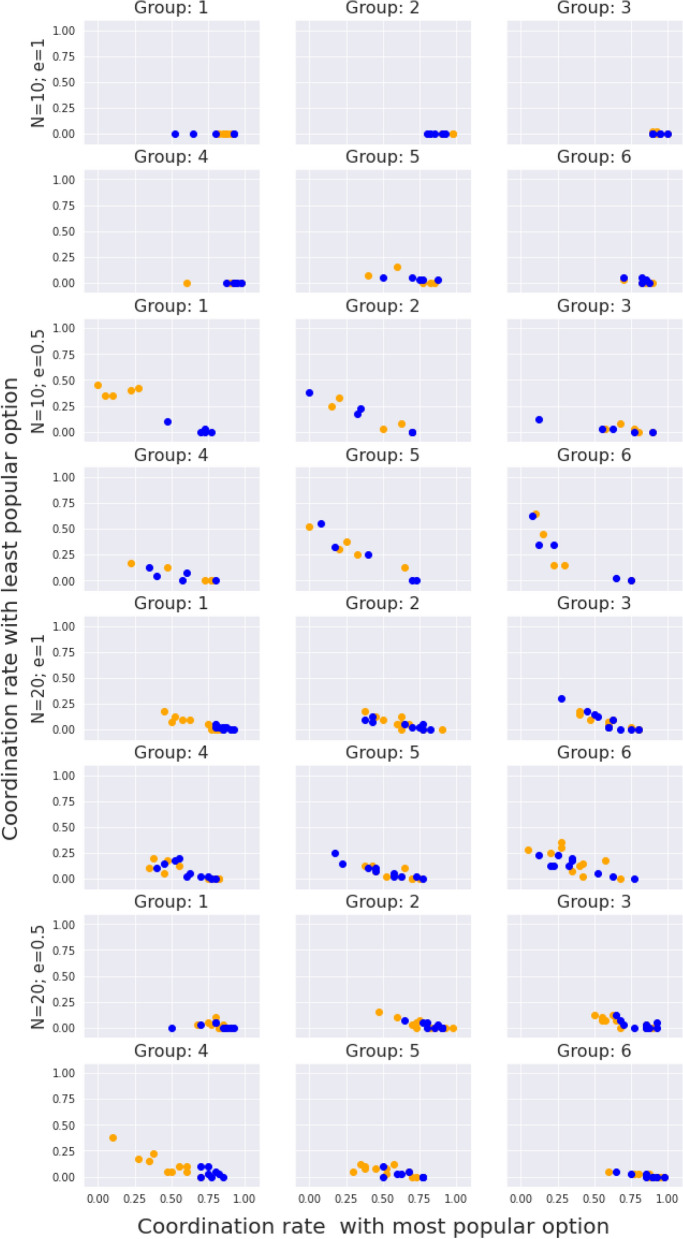
Experimental profiles of coordination rates for the transient (rounds 0 to 40); X-axis: coordination rate within the subpopulation that shared the most popular action in the population; Y-axis: coordination rate within the subpopulation that shares the complementary action. Coloured, the marker of the participants.

The results of this analysis are plotted in Fig. 2, which shows different types of behavior on different groups. Some groups, like groups 1, 2, 3 in the configuration with $\{N=10,e=1\}$, have all their points lying in the horizontal axis. This indicates a collective prevalence of the popular opinion, in agreement with the fact the collective equilibrium has arisen independent of the markers. There are also groups that show a coordination distribution around a line with negative slope, like groups 2 and group 5 from the configuration $\{N=10,e=0.5\}$. This means that the popular opinion induces a better coordination rate, and that the herding behavior is not as strong, as the other opinion attracts some coordination. This situation may be related with a marked behavior, as in group 1 of $\{N=10,e=0.5\}$, but does not have to, as in group 2 from the same configuration. In the first case, subgroups arranged by markers arrive to two different consensus, and then, one starts to become dominant. In the second case, we are just in a transition moment towards the case analyzed in the previous paragraph. In any case, we can conclude that Fig. 1 showed that marked behavior does not produce different agreements between marked groups, but Fig. 2 shows that the final unique consensus may be a result of a competition process between marked groups. Furthermore, this marked behavior in the discussion process towards choosing an unique consensus is just a possible qualitative option. A global consensus that do not include markers is always a possible option.

**Figure 3:**
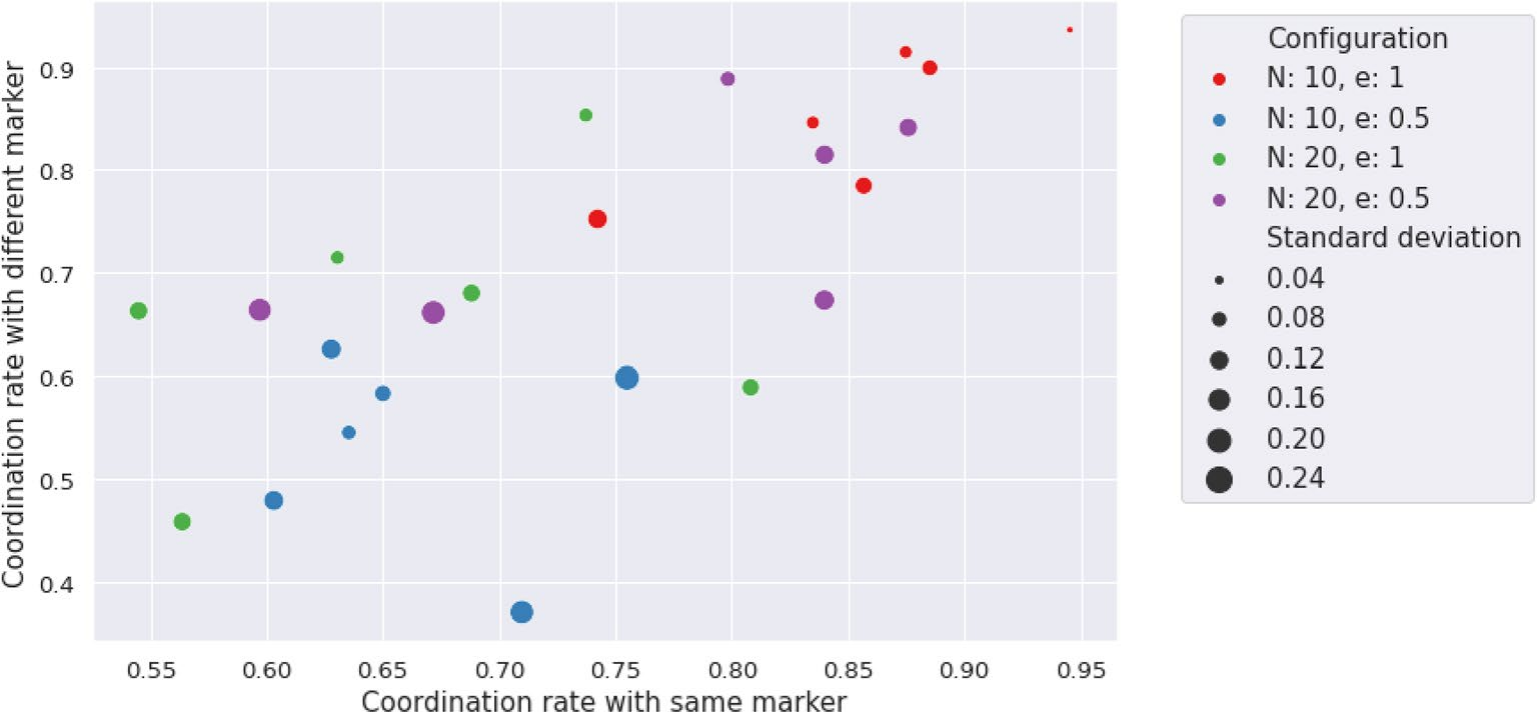
Coordination with agents with same and different marker for different *e* and group size. Groups are characterized by four different configurations according to these parameters: $\{N,e\} = \{\{10,0.5\},\{10,1\},\{20,0.5\},\{20,1\}\}$.

**Figure 4:**
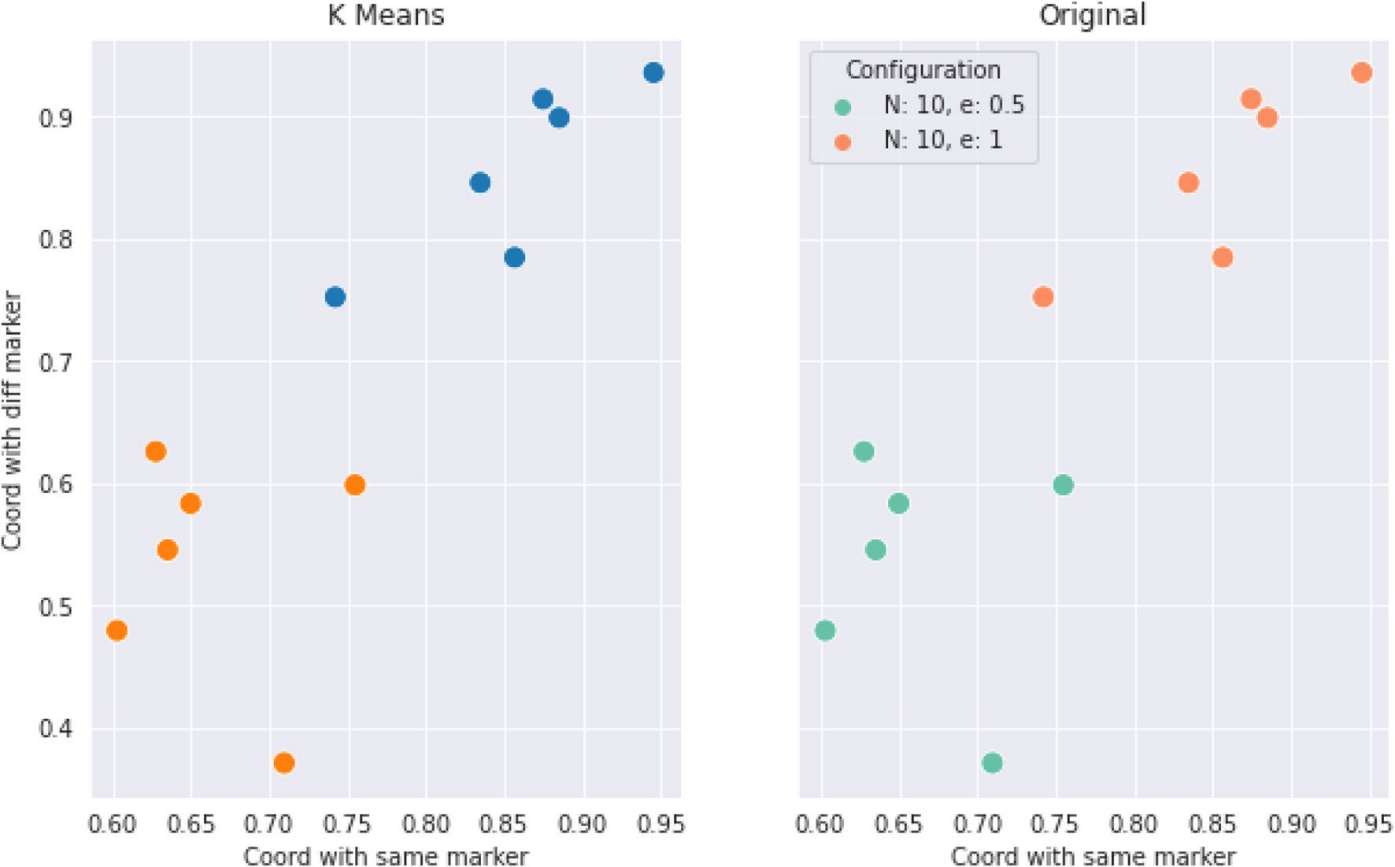
K-Means clustering for the groups that have a size of $N=10$ participants. The separation confirms that the space of parameters of coordination rate separates the groups.

**Figure 5:**
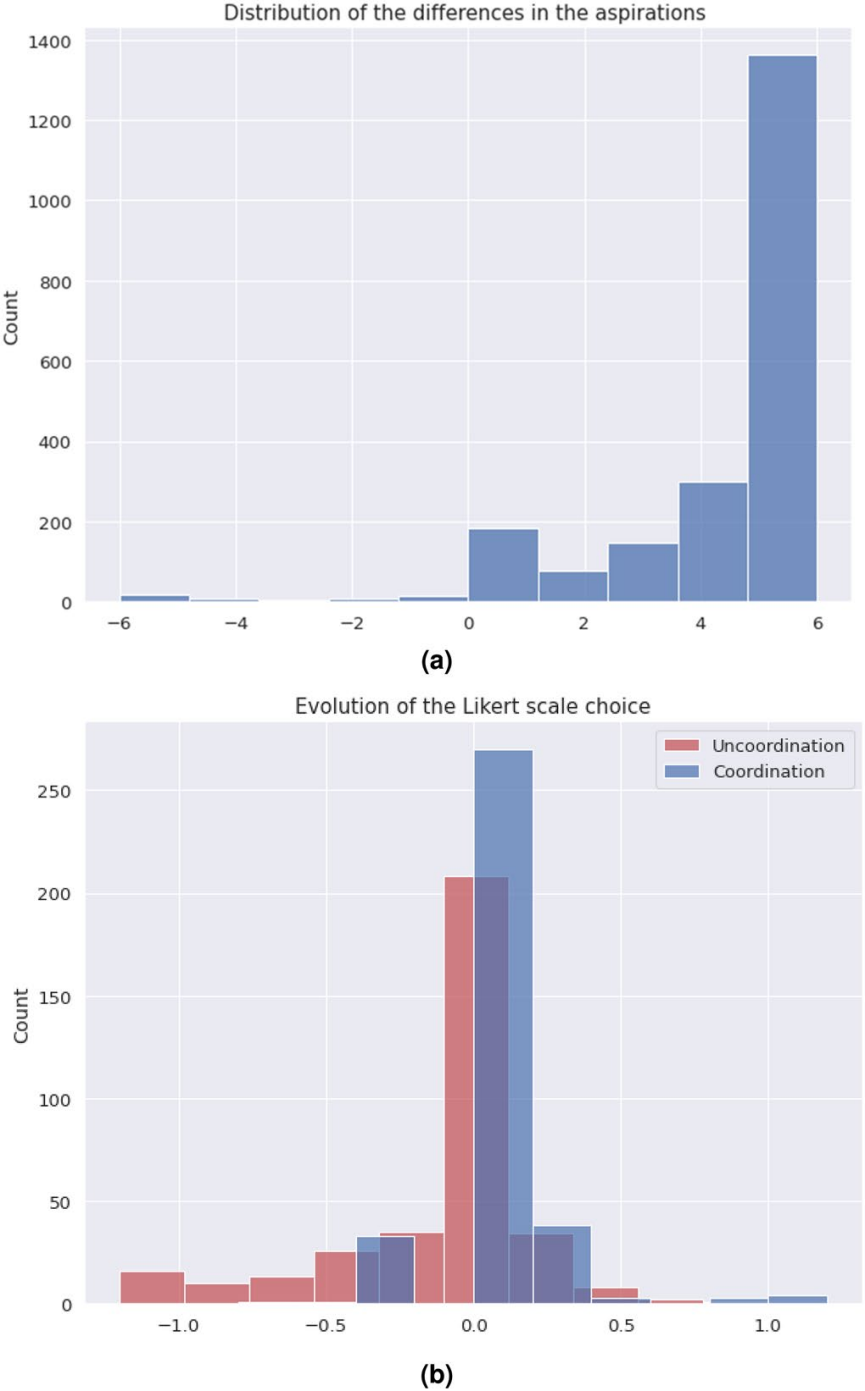
(**a**) Distribution of the difference between the valuation of the outcomes. (**b**) Distribution of the temporal differences between the different times participants were asked about their valuation.

**Figure 6:**
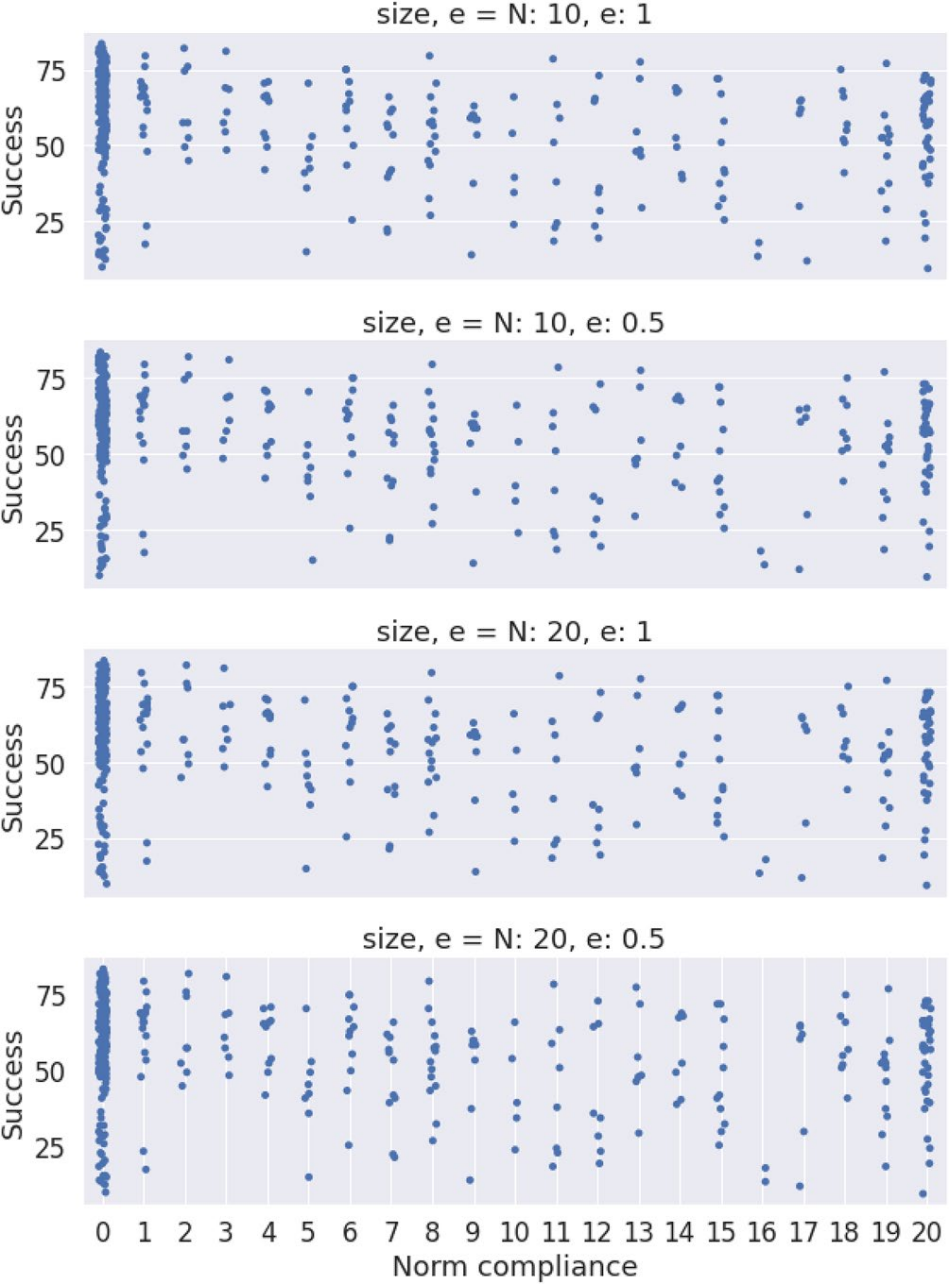
Relation between the binary coordination game and the degree of norm compliance (measured from 0 to 20). On the X axis, the degree of norm compliance; on the Y axis, the number of coordinations for a player during the game.

**Figure 7:**
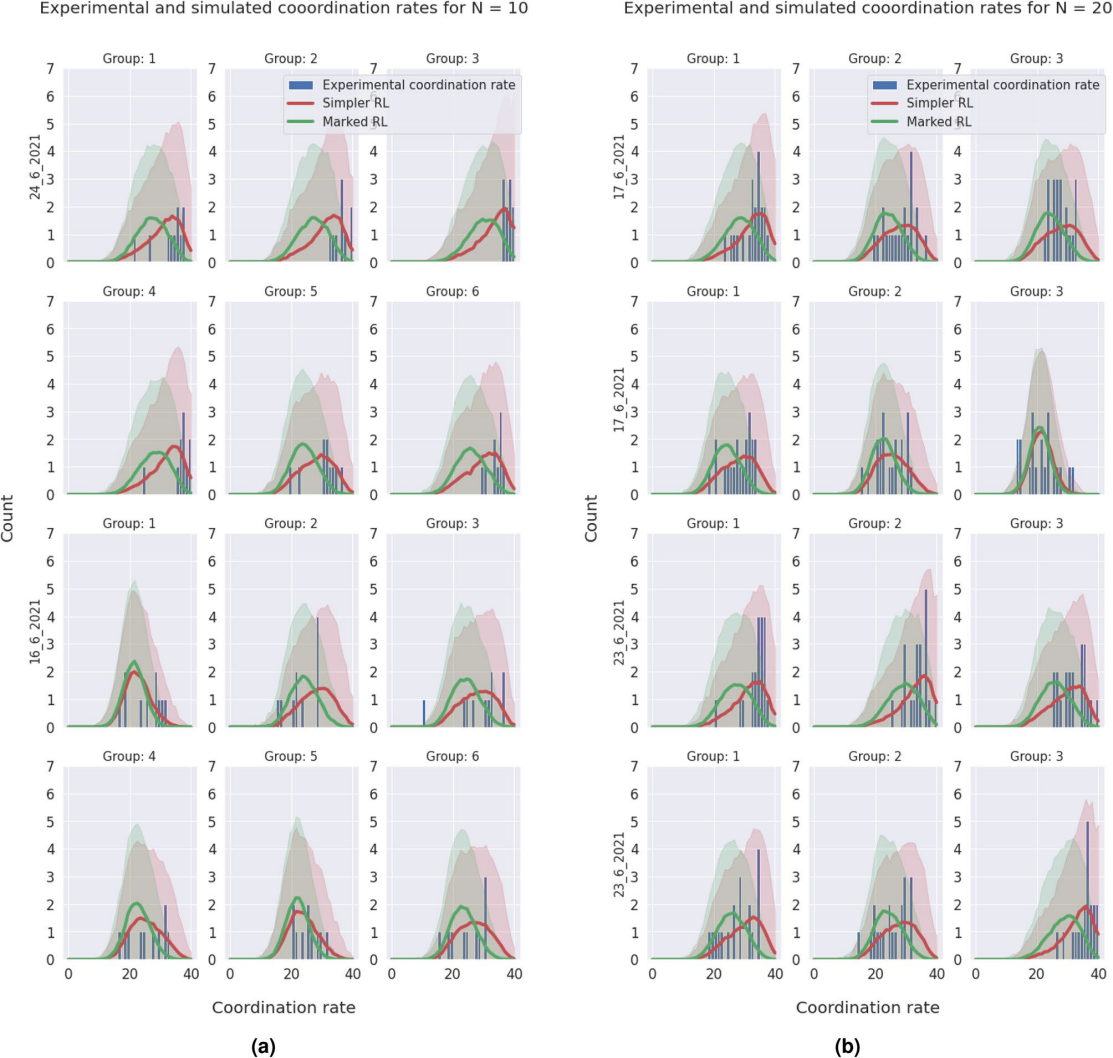
(**a**) Coordination rate distribution for the different populations with $N=10$ for the rounds (0, 40). (**b**) Coordination rate distribution for the different populations with $N=20$ for the rounds (0, 40).

**Figure 8:**
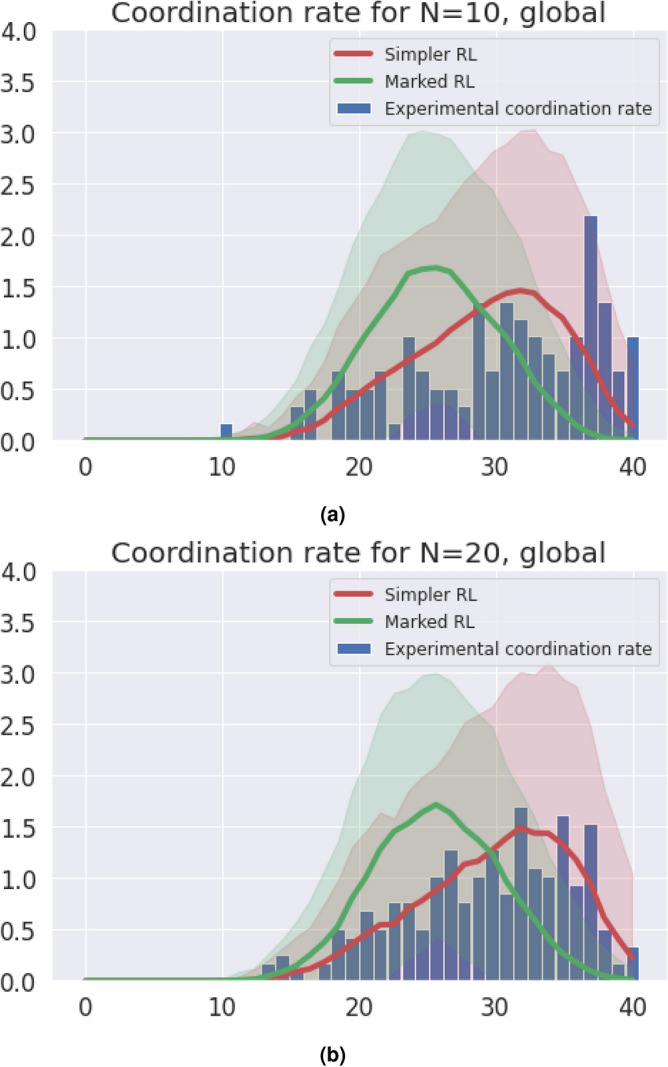
(**a**) Coordination rate distribution for the sum of the populations with $N=10$ for the rounds (0, 40). (**b**) Coordination rate distribution for the sum of the populations with $N=20$ for the rounds (0, 40).

In order to better understand our results, we set out to reproduce the experimental results with a simplified version of our original agent based model^7^. That model was based on a reinforcement learning algorithm that took into account different strategies for the different markers. Now, to incorporate the fact that markers do not seem to guide the actions of our real players, we studied one thousand simulations of a simplified model where agents have only one strategy to update, which they use against any opponent irrespective of their marker. We did this for each group, tuning the learning parameter to the experimental observations, obtaining the results shown in Fig. 7a,b for the distribution of the coordination rate. The thick line shows the statistical average and the shadowed region is twice the standard deviation of the simulations. It is clear from the plot that the simpler model is a better description of the experiments than the original one.

To provide less noisy results, we also studied the results for each group in Fig. 8a,b, joining together all the groups with the same group size and homophily parameter. In this case, we tuned the learning rate simply choosing average of the ones that have been tuned in the former step, i.e., for the individual groups. Here, as we have a bigger sample, we only plotted one standard deviation as the shadowed region. As we can see, the statistical properties of the coordination distribution can again be better reproduced by the model without markers. This results, together with the absence of domination of marked behavior in our experimental results and in the question of perception, drives us towards recognizing that markers are not equilibrium separators. Populations tend to choose more simpler strategies, and in a coordination game, the simplest one is a single action for everyone.

The fact that our main hypothesis, namely the appearance of correlations between the opponent’s marker and the behavior chosen by the focal agent, is not confirmed in most cases prevents us from validating the rest of our hypotheses. Indeed, all of them discuss to some extent features of the behavior-marker correlation, and we have not observed it in the experiments. Nevertheless, in what follows we continue to present our results in terms of our hypotheses analyzing what happens with the convergence to a uniform, global behavior: i.e., we consider convergence to equilibria on the same action irrespective of the marker of the opponent. This will allow us to extract insights from our experiment which can be useful in order to improve theoretical models or to design further experiments to search for the correlation we have not observed.

In the above spirit, let us now discuss the hypotheses referred to the velocity of the system towards equilibrium (this is, **H2** and **H5**), we used the situation at the end of the transient as a checkpoint in the coordination consensus. To compare the outcome of the experiments with different parameters, we focus on the average coordination at the end of the transient (which, from now on, we define to be the first forty rounds). We will also refer to the behavior adopted by all the agents at the end of the experiment as the final equilibrium. It goes without saying that we cannot be sure that this behavior remains unchanged forever, but we think it is quite unlikely that it changes because there is not any incentive for a player to deviate from coordinating with everybody else, whereas it does not seem plausible that a group of players that cannot communicate could simultaneously modify their behavior. This magnitude allows us to compare all groups at the same timestep and check the dependencies on group size and homophily. To this end, we evaluate the frequentist probability of coordination at the end of the transient for each individual in a group, then we take the group average, and subsquently compare the distributions of the different group averages.

To present this comparison of the convergence to equilibrium for different parameters (group size and homophily), we resort to a single plot, namely Fig. 3. We used a scatter plot to show the distribution of these group averages for the different configurations, along with its standard deviations expressed in the size of the circle. The homophily parameter can be interpreted using the sign of *“Configuration”* in the legend. A positive sign means *e = 1* and a negative one means *e = 0.5*. The number indicates the group size. So, for example *20* indicates a configuration of 20 agents with a parameter *e=1*.

From Fig. 3, it can be checked that there is no correlation with any group size nor homophily parameter, as there is no ordering but overlap between distributions. In fact, in can be checked that the levels of coordination are higher for a group size of 10 and *e = 1*, together with size of 20 and *e = 0.5*, showing a decorrelation between these variables.

**Figure 9:**
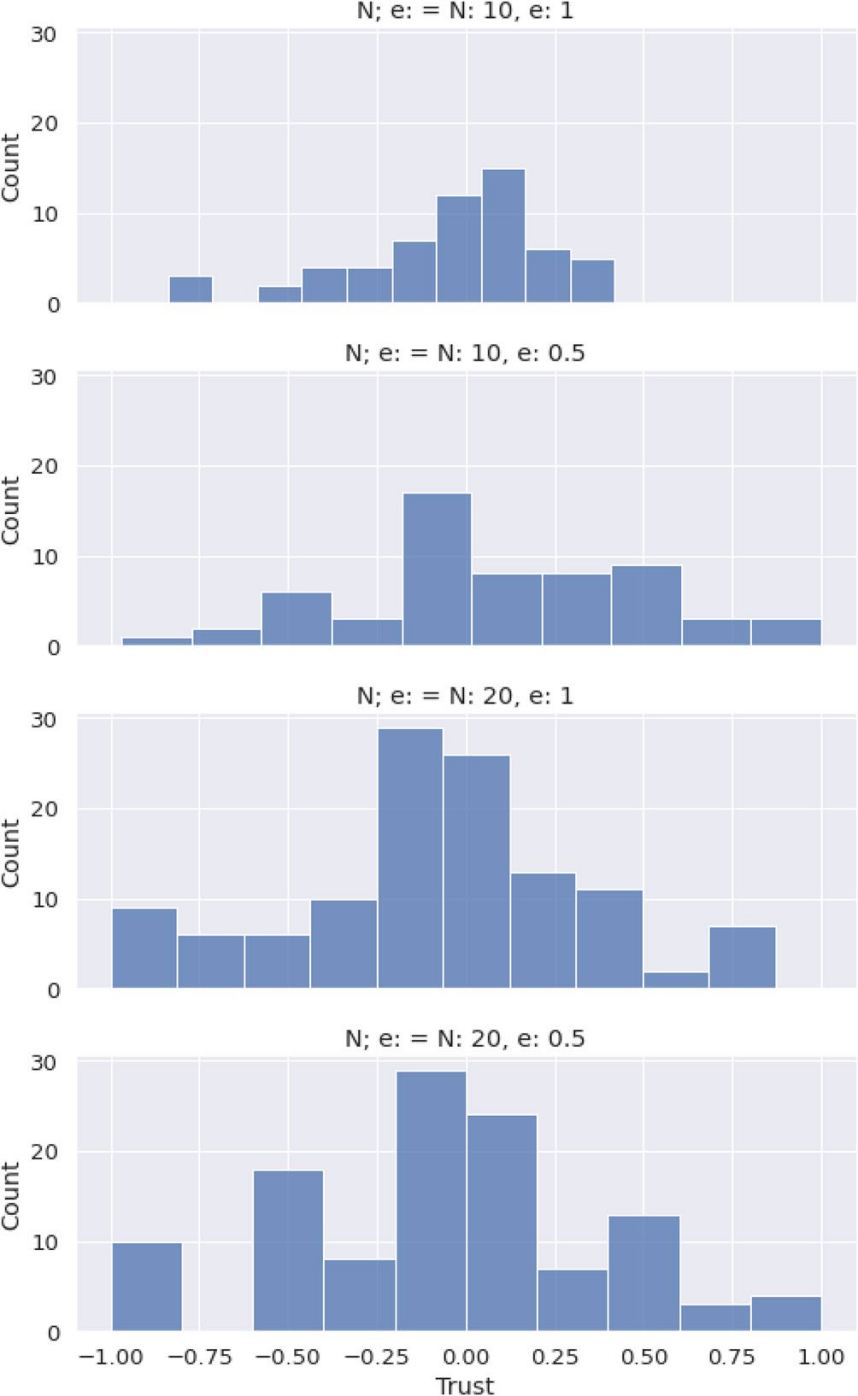
Experimental profiles of trust distributions for the four different configurations, depending on the parameters *N,e*.

With this information we can conclude the following:

- Hypothesis **H1** is not confirmed, as marked behaviors are only occasionally observed. The general behavior does not exhibit correlation between the marker of the opponent and the action the subject chooses. The preferred option by the subjects is an homogeneous equilibrium towards a single consensus action.
- Hypothesis **H2** is also not confirmed, as it derives from the previous one. The statement about homophily saying that it increases the creation velocity of the intra-marker correlation, this is, the action of individuals that share marker is wrong because, even if we consider the general consensus instead of an intra-marker correlation, homophily does not correlate with coordination.
  In order to check hypotheses **H3** and **H4**, that are related to the preferences of the participants, we use data from the Likert scale questions mentioned in the methods section, and we plot a histogram of the difference between the valuation of coordination and of uncoordination. In this histogram we aggregate data for all the times participants were questioned about their assessment, yielding Fig. 5b. From a total of 2448 answers, 92% of the times the coordination payoff was valued the highest. From the remaining 8%, only a 1.5% (5 players out of 360) consistenly regarded higher the uncoordination outcome. Likewise, the time evolution of these answers was measured via the average of the differences in the Likert scale between consecutive replies for each player. Figure 5a presents a histogram of the distribution of these averages, showing that these differences are not statistically relevant, supporting the hypothesis of the stationary assesment. Therefore we can state that:
- Hypothesis **H3** can not be confirmed according to the information of the experiment. It is worth mentioning that this hypothesis is always conditioned by hypothesis **H1**, meaning that the correlation we are referring to in this hypothesis is not any marked correlation, but the unique action strategy. This being said, it is true that this strategy exists always and that in all experimental sessions a vast majority chooses the preference order we pointed out in the hypothesis. Nevertheless, we can not say this is the main factor in order to reach this equilbrium, although both facts are correlated in the experiment.
- Hypothesis **H4** shows a similar problem as hypothesis **H3**, in the sense that the correlation we are referring to has to be the unique action strategy. Taking this into account, the preference order is stationary and the qualitative result is this single cluster of actions. We can not confirm the direction of the implication, but it can be said that both things are correlated in our experimental sessions.
- Hypothesis **H5** can not be verified. As can be observed in Fig. 3, the average coordination is not correlated with the group size. Although there is no full separation between the groups from different configurations, we have sliced the data for $N=10$ and made a *k*-means clustering with $k=2$, trying to separate the homophily configurations. As it can be checked in Fig. 4, the algorithm is capable of splitting correctly both subpopulations. We can conclude that within the uncertainty about hypothesis **H5**, we can separate accurately the homophily configurations within a certain group size.
  Hypotheses **H6** and **H7** are related to the strength of the marked correlations and the degree of norm compliance. We measure the norm compliance using a different game described in the “Methods” section. The main result of this interaction is shown in Fig. 6 where we plot the correlation between the number of coordinations in the first game vs the degree of norm compliance in the second game(measured in the number of yellow boxes the player has chosen, see “Methods”).
- Hypothesis **H6** can not be confirmed. Firstly, it derives again from hypothesis **H1**, which proposes marked-related behavior as a possible solution to the coordination problem. But besides this, if we think the equilibrium in terms of a consensus solution, norm compliance is not correlated with a capacity to reach a better result in the coordination game.
- Hypothesis **H7** is wrong as both processes are independent. In particular, the norm compliance game exhibits a trend towards a bimodal process, where agents can be mainly split into a group which complies and a group of conformists.
  Finally, to test hypothesis **H8**, we used a perception question, in addition to the binary coordination game and the norm compliance test, to guess the affinity of participants with other participants sharing their marker. We asked for the number of dots in a random image and offer two answers, each one characterized by a marker. Then, we asked them for a number (see “Methods”). We define $\Delta m_{=}$ as the distance between the correct number of points and the answer labeled with their own marker and $\Delta m_{\ne }$ as this distance with the other marker. Then , we define:
  4 $$\begin{aligned} t(m) = \dfrac{\Delta m_{=} - \Delta m_{\ne }}{\Delta m_{=}+\Delta m_{\ne }} \end{aligned}$$
  a quantity we have called trust. If both distances are similar, $t \rightarrow 0$; and if one of the distances is 0, then it goes either to + 1 or − 1. A marked behavior would make this behavior more salient. We plotted the distribution of this quantity for the different populations in Fig. 9. The significance of both differences in distance can be computed using a Wilcoxon test on the trust distribution. We obtained a p-value of 0.19, which leaves the hypothesis that groups can be differentiated unsustained. Both groups can be understood as the same, so the fact that the marker is shared by the subject is not relevant.
- Hypothesis **8** can not be subsequently validated, as individuals are not influenced by others who share their markers when making decisions, as the distributions from Fig. 9 prove.

Collecting all the results above, we can conclude that, given that the existence of the markers does not play an important role in our experimental results, when we think of our hypotheses in terms of the dynamics of the global coordination process, they are not sustained. Of course, it has to be borne in mind that this does not mean that they are disproven in contexts in which markers could indeed induce different behaviors.

## Conclusions

As we have seen, marked behavior, i.e., behavior that takes into account the marker of the opponent, is not the usual outcome observed in our experiments. Indeed, we observe that markers have a limited role both in space and time. During the transient and the first rounds, homophily sometimes helps to reach a separated consensus for the subpopulations with the same marker, but even in these cases, one of the actions tends always to become the dominant one, pushing the equilibrium configuration towards a single action. Once we have established this result, we have focused on the dynamics of the global coordination process, a context we have tried to understand following the guidance of our preregistered hypotheses. In this manner we have observed that global coordination does not appear to be well described by those hypotheses. On the other hand, the influence of external parameters such as group size or homophily on these qualitative results is not quantitatively important, and we do not find evidence for marked behavior for any choice of parameters except perhaps for the case of small groups. Finally, using computational models, it can be checked that these experimental results may be reproduced using a non-marked model reinforcement learning dynamic, supporting our conclusions.

As we have shown, the results of our experimental sessions show that binary markers, understood by the players as symbolic “hats”, seem not to lead generally to consensus on different actions for different markers in a coordination game. This observation is independent of the group size and other parameters related to the selection rules of the subjects such as homophily. Of course, this conclusion is limited to our specific experimental set up, designed to mimic computational models^7,11^. In particular, an important point to keep in mind is that in our experimental setup, as well as in the two theoretical models we are comparing our results with, there is no initial correlation between markers and behaviors. According to the calculations in Ref.^11^, some small marker-behavior covariance may be needed to initiate the evolutionary process leading to people with different markers choosing different behaviors. Interestingly, they also find that if this covariance is large, the effect may be less pronounced, somewhat counterintuitively, which leads them to introduce migration in their model (not included in our experiment). It is then possible that if we had started by directing or suggesting participants with a given marker to try a specific behavior then the correlation would have set in and become stable. That would certainly support the idea that the markers we are considering may support different equilibria or norms in different groups, but we would still be left as to the origin of the first seed for such coexistence. The discussion in Ref.^11^ seems to indicate that migration from geographically separated groups where this covariance already existed could lead to the correlation between behaviors and markers, but this brings up the question of its emergence in the first group.

We stress that our results do not mean that external visible information does not play an important role in the functioning of society, as it has been argued in the introduction of the paper. Sociological evidence is clear about simplifications and symbolism that help people take decisions in a fast and efficient way. However, our experiment indicates that the complexity that lies behind these symbols is larger than the one we have used in this experiment. We intended to create a marker without significance on purpose to analyze this innocuous information as a spontaneous symmetry breaker of the population into groups. In this context, meaning in the conditions of the random binary visible information we assign to the participants, the “force of a simpler criteria” (namely, coordinate in the same action as everybody) is apparently stronger than the possibility to break the group into several cells with different behaviors. However, this may not be true when we are talking about decisions that contain a personal or sociological meaning, like the ones inherent to culture. When people has a preference order that can classify external information according to their individual preferences, it is clearly possible that more complexity can arise from this type of set ups. This important question merits further research with experiments that include markers in a more natural or sociologically relevant form.

## Methods

Experimental sessions were implemented in IBSEN-oTree. The participants played in a web browser in a phone, computer or tablet. Informed consent was obtained from all the participants through the IBSEN volunteer pool. Anonymity was preserved, according to the Spanish Law for Personal Data Protection. This procedure was approved by the Ethics Committee of the University Carlos III, the institution that founded the experiment. The experiment took place according to the corresponding guidelines.

A total of *360* subjects were recruited distributed in six different experimental sessions in June 2021. Each experimental session was formed by 60 subjects divided in several groups. We tuned two different parameters, the homophily (*e* = 1 and *e* = 0.5) and the size of the population (*N* = 10 and *N* = 20). For each of these four different configurations, we aimed to study six of these populations of size *N*. Once the experimental session starts, and after signing the consent and reading the instructions, each agent was informed about their marker. Then they played the binary coordination game. For each round, agents were randomly paired and had to take one of two actions. If both actions coincide, both players receive two points; if the actions are different, both players diminish their total payoff by one point. The minimum payoff an agent can have is zero points. At the beginning and every 15 rounds, a question about categorizing the payoffs of the game is done. Given a Likert scale from 1 (worst) to 7 (best), participants are asked about how do they feel about getting each of the payoffs. After 75 rounds, agents were asked two types of questions. A first one, related to marker affinity, where we asked about how many dots there were on a image, with a total number of 653. We offered two answers to the participants, and inform them that each of the answers had been answered by other participants, each one with a different marker. Our goal is to measure how prone are the participants about these answers based on their markers. After these questions, we included a second game related to norm compliance. This game consists in including balls in two different boxes. One box provides twice as payoff as the other one, but in the instructions participants are told to put all the balls in the box with the minimum payoff. The game lasts for 20 rounds and we are trying to measure the susceptibility of the participants.

In the following we provide a translated version of the instructions given to the participants in the two games:

- **Ethnic markers game:** This first part consists in several rounds. Before starting, you will be asigned a coloured hat, which will stay the same for every round. Your hat will be visible for all the participants. Neither the hat nor its colour are related with any pay. Every fifteen rounds you will be asked two brief questions. After that, you will be paired with another participant, which can change every round. You will be allowed to see your couple’s hat. In each round, you and your couple should choose between two actions. If both choose the same action, you will receive a payoff of 1 point. If the actions are different, you will receive a − 0.5 payoff. You can not have negative points, so the minimum punctuation is zero points. You can not communicate with your couple.
- **Norm compliance game:** In this second part, you will be asked to choose a box, either blue or yellow, where you should deposit twenty balls. You will have ten seconds for each decision. For every ball you put in the yellow box, you will be rewarded with 0.5 points. For every ball you put in the blue box, you will be rewarded with 1 point. The norm is that balls must be put in the yellow box.
